# Ein seltener Grund für rechtsseitige Oberbauchschmerzen

**DOI:** 10.1007/s00104-022-01625-8

**Published:** 2022-03-31

**Authors:** Anna Schütte, Linda Dießel, Martin Stockmann, Jens Walldorf

**Affiliations:** 1 Universitätsklinikum Halle (Saale), Ernst-Grube-Str. 40, 06120 Halle (Saale), Deutschland; 2 Evangelisches Krankenhaus Paul Gerhardt Stift, Paul-Gerhardt-Straße 42, 06886 Lutherstadt Wittenberg, Deutschland

## Anamnese

Eine 52-jährige Patientin stellte sich wegen seit zwei Wochen zunehmender rechtsseitiger Oberbauchschmerzen vor. Eine Cholezystolithiasis sei bekannt, aber bisher asymptomatisch gewesen. Postprandial sei es zu einer Verstärkung der Symptomatik gekommen. Fieber habe nicht bestanden, ebenso kein Ikterus, aber – ebenfalls seit etwa zwei Wochen – Schüttelfrost, Inappetenz und Nachtschweiß. Bisher sei die Patientin immer gesund gewesen. Vor zwei Jahren habe die Patientin in Thailand Urlaub gemacht.

## Klinischer Befund

Bei der körperlichen Untersuchung fand sich ein weiches Abdomen mit deutlichem Druckschmerz im rechten Oberbauch ohne Abwehrspannung. Sonographisch wurde eine vergrößerte, in Richtung Leber wandverdickte Gallenblase mit multiplen Konkrementen sowie ein beginnender Abszess gesehen, außerdem eine geringe Menge Aszites, betont perihepatisch, mit winzigen peritonealen Auflagerungen.

## Aufnahmelabor

Hämoglobin 7,7 mmol/l, Leukozyten 5,12 Gpt/l, CRP 0,6 mg/l, auch die weiteren Parameter (insbesondere Differenzialblutbild, ASAT, ALAT, AP, GGTP und Bilirubin) waren unauffällig.

## Weiteres Prozedere

**Figure 1:**
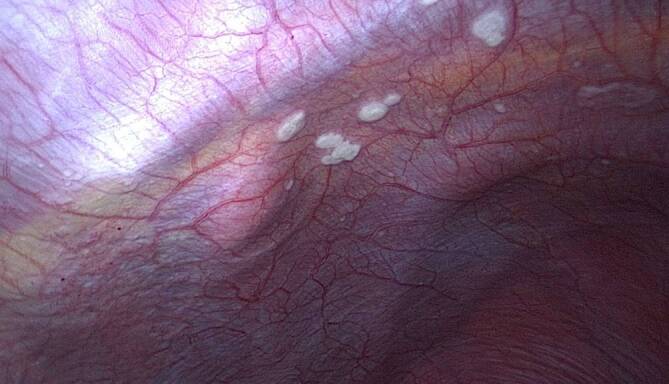

Bei B‑Symptomatik und sonographischem Bild von Leberraumforderungen mit Peritonealkarzinose erfolgte zunächst eine diagnostische Laparoskopie. Hier zeigten sich über 20 peritoneale Herde sowie weitere Läsionen an der Serosa des Magens, Kolons und Omentum majus (Abb. 1). Es erfolgten eine parietale Peritonektomie, ein Débridement des Leberabszesses mit Lavage und Drainage sowie eine Stanzbiopsie im Lebersegment 6.

**Figure 2:**
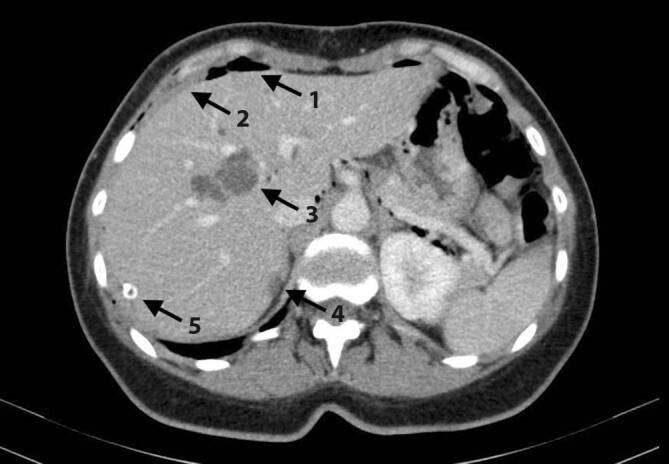

In der postoperativ angefertigten Computertomographie (CT) von Thorax und Abdomen (mit i.v. Kontrastmittel) zeigten sich hypodense Läsionen der Leber in Segment 6, 7 und 8, zystische Läsionen in Segment 4a sowie peritoneale Auflagerungen perihepatisch (Abb. 2). Weitere abdominelle oder pulmonale Manifestationen wurden ausgeschlossen. Die weiterführende Diagnostik inklusive Ösophagogastroduodenoskopie, Koloskopie und gynäkologischer Vorstellung ergaben keinen Anhalt für weitere Pathologien.

## Histologie

**Figure 3:**
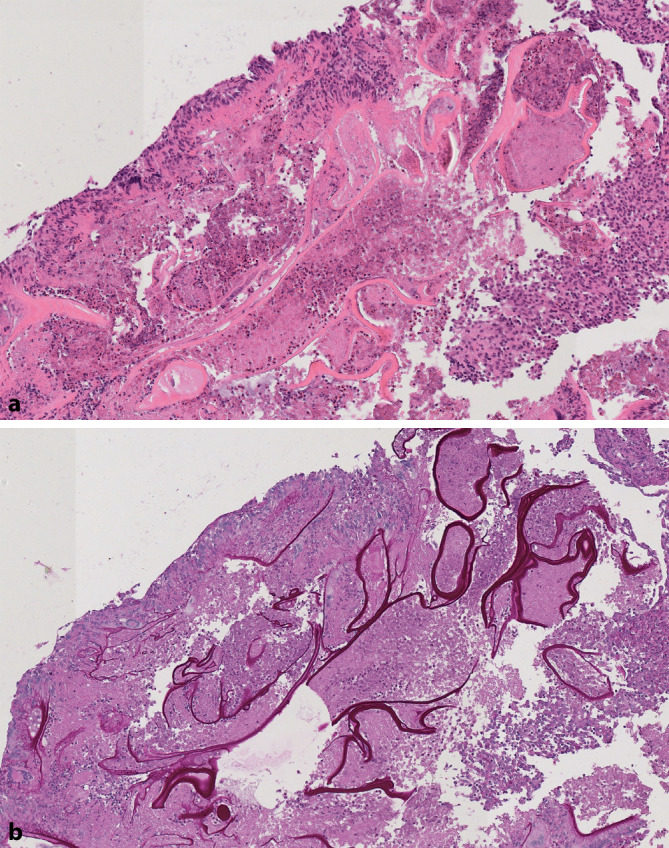

In den Biopsien aus dem Peritoneum und Leberabszess zeigten sich Nekrosezonen und Granulationsgewebe, dichtes entzündliches Infiltrat mit fokaler Abszedierung und zahlreichen eosinophilen Granulozyten sowie randständig palisadenartige Ansammlungen epitheloider Histiozyten neben Fibrininsudationen. Innerhalb des entzündlichen Infiltrates ließen sich PAS(„periodic acid-Schiff“)-positive Membranen darstellen (Abb. 3), jedoch keine atypischen Proliferationen.

Die Leberbiopsie zeigte reguläres Leberparenchym.

## Wie lautet Ihre Diagnose?

## Definition

Zur Bestätigung der Diagnose erfolgte eine immunologische Diagnostik. Hier waren Echinokokkenantikörper nachweisbar; mittels Enzym-linked Immunosorbent Assay und Westernblot wurde eine alveoläre Echinokokkose (*Echinococcus mulitlocularis*) bestätigt.

Der Hauptwirt des *Echinococcus multilocularis* (Fuchsbandwurm), dem Erreger der alveolären Echinokokkose, ist der rote Fuchs, Zwischenwirt sind Nagetiere und auch Haustiere wie Hunde und Katzen. Vor allem Hunde, die infizierte Nagetiere fressen, sind für die Übertragung auf den Menschen von Bedeutung [1]. Bei Landwirten ist die Diagnose als Berufskrankheit anerkannt. Der Erreger findet sich fast ausschließlich auf der nördlichen Erdhalbkugel, in Europa ist der Erreger in der Alpenregion hochendemisch. In Deutschland wurden in den letzten 10 Jahren der Statistik der Robert-Koch-Institutes zufolge jährlich im Durchschnitt 41 Fälle einer alveolären Echinokokkose gemeldet (zystische Echinokokkose 75 Fälle/Jahr; [2]), wobei von einer großen Dunkelziffer auszugehen ist [3].

Aufgrund einer Inkubationszeit von 10 bis 15 Jahren gelingt eine Diagnosestellung in der Regel sehr verzögert und der genaue Übertragungsweg kann nicht mehr nachvollzogen werden [3]. Initiale Herde finden sich fast immer in der Leber, im Verlauf können weitere Organe betroffen sein. Analog dazu umfasst die Symptomatik zu Beginn häufig abdominelle Beschwerden, Ikterus, Gewichtsverlust, Fieber und Anämie. Bei progredienter Erkrankung sind auch eine Leberdysfunktion mit portaler Hypertension möglich [1]. Blutbildveränderungen wie eine Eosinophilie finden sich nur bei unter 25 % der Patienten [4].

Bildgebend finden sich typischerweise unregelmäßig begrenzte, in der Sonographie echogene bzw. in der CT hypodense Läsionen mit zentraler Nekrose und Kalzifikationen; eine intrahepatische Gallengangserweiterung kann mitunter beobachtet werden [3]. Als Screeninguntersuchung der Wahl hat sich die B‑Bild-Abdomensonographie etabliert, die durch die kontrastverstärkte Sonographie ergänzt werden kann. Als schnittbildgebendes Verfahren gilt die Computertomographie als Methode der Wahl, insbesondere zur Darstellung von Kalzifikationen und zur Metastasensuche. Die Diagnosestellung bleibt auch nach bildgebender Diagnostik herausfordernd, insbesondere die Abgrenzung gegen maligne Veränderungen ist oft unmöglich. So erfolgte auch in diesem Fall beim Verdacht auf eine fortgeschrittene maligne Erkrankung zunächst eine diagnostische Laparoskopie.

Immundiagnostische Tests sind zur Diagnosestellung erforderlich: Zum Screening wird auf das *E. granulosus*-Hydatidenflüssigkeits-Antigen (EgHF-Ag) und das *E. multilocularis*-Vesikelflüssigkeits-Antigen (EmVF-Ag) getestet, des Weiteren stehen *E. multilocularis*-spezifische ELISA-Tests zur Verfügung. Die Sensitivität dieser Diagnostik liegt bei ca. 95 %. Zur weiteren Differenzierung und Abgrenzung des *E. mulitlocularis* gegen *E. granulosus* erfolgt die Testung mittels Westernblot [1].

Gegebenenfalls kann eine Leberherdbiopsie oder explorative Laparotomie zur histologischen und molekularen Sicherung bzw. Ausbreitungsdiagnostik durchgeführt werden [4]. Bei der Zystenpunktion muss im Falle einer alveolären Echinokokkose das Streuungsrisiko beachtet werden [1]. Histologisch beweisende Protoskolizes sind nur selten sichtbar, typischerweise finden sich allerdings PAS-positive lamellenartige Membranen [3].

Unbehandelt verläuft die Erkrankung tödlich (Mortalität nach 10 Jahren 90 %, nach 15 Jahren 100 %). Im Frühstadium ist eine Perizystektomie gefolgt von mindestens 2 Jahren medikamentöser Behandlung mit kurativem Ansatz möglich, je nach Ausdehnung des Befundes können ausgedehntere Resektionen (Hemihepatektomie, atypische Resektionen) erforderlich sein, um alle betroffenen Areale zu entfernen („R0-Resektion“). Im fortgeschrittenen und inoperablen Stadium ist eine lebenslange Therapie mit Albendazol oder Mebendazol indiziert, um das Wachstum der Larven zu hemmen, die Metastasierung zu reduzieren und die Lebensqualität und -länge zu verbessern [4]. Bei Patienten mit fortgeschrittener alveolärer Echinokokkose unter alleiniger medikamentöser Therapie findet sich ein 15-Jahres-Überleben von über 80 %. Im Gegensatz zur Behandlung der zystischen Echinokokkose (*E. granulosus*) hat die chirurgische Therapie bei der fortgeschrittenen alveolären Echinokokkose einen begrenzten Stellenwert: Der Nutzen einer palliativen chirurgischen Therapie („debulking“) in Kombination mit medikamentöser Behandlung ist fraglich. Vereinzelt wurden Fälle beschrieben, in denen eine „neoadjuvante“ Langzeittherapie mit Albendazol oder Mebendazol eine Leberresektion bei initial inoperablem Stadium möglich machte; in ausgewählten Fällen wurde eine Lebertransplantation durchgeführt – mit allerdings wenig ermutigenden Ergebnissen [5]. Inwieweit diese Optionen bei einer peritonealen Streuung der Infektion – wie im hier geschilderten Fall – sinnvoll sein können, ist unklar. Der Goldstandard für diese Situation bleibt die medikamentöse Dauertherapie.

## Therapie und Verlauf

Im hier geschilderten Fall einer Infektion mit *Echinococcus multilocularis* war aufgrund der peritonealen Streuung („Metastasierung“) eine chirurgische kurative Therapie nicht möglich. Es wurde eine lebenslange Therapie mit Albendazol 2‑mal 400 mg per os unter regelmäßiger Kontrolle der Transaminasen und Cholestaseparameter empfohlen. Vor dem Therapiebeginn erfolgte zur Komplettierung der Diagnostik eine Computertomographie des Kopfes zum Ausschluss zerebraler Herde.

**Diagnose:** metastasierte alveoläre Echinokokkose

Unter der Therapie mit Albendazol klagte die Patientin zunächst über Übelkeit und abdominelle Beschwerden, laborchemisch zeigte sich ein geringer Anstieg der Transaminasen. Zunächst wurde die Therapie unter engmaschigen Laborkontrollen in gleicher Dosierung fortgeführt. Im Verlauf zeigte sich eine deutliche Besserung des Allgemeinbefindens der Patientin, die Transaminasen normalisierten sich und die sonographische Kontrolle nach einem und nach vier Monaten zeigte einen stabilen Befund der Leberläsionen.

## Fazit für die Praxis

- Aufgrund der langen Inkubationszeit und der unspezifischen Frühsymptomatik ist die Anamnese meist nicht wegweisend.
- Unklare Leberläsionen mit Zysten, Verkalkungen oder zentraler Nekrose sollten an eine Echinokokkose denken lassen und zu entsprechender immunologischer Diagnostik führen.
- Die Therapie erfolgt kurativ durch eine Perizystektomie bzw. Leberresektion mit adjuvanter medikamentöser Therapie oder bei fortgeschrittenem Befund durch lebenslange Gabe von Albendazol oder Mebendazol.
